# Supplementary material for: Reconciling Mining with the Conservation of Cave Biodiversity: A Quantitative Baseline to Help Establish Conservation Priorities
Source: PLoS One. 2016 Dec 20;11(12):e0168348. doi: 10.1371/journal.pone.0168348 (PMC5173368; doi:10.1371/journal.pone.0168348)
Supplement: S1 Dataset — (ZIP) [file pone.0168348.s002.zip › Taxa/Serra Norte/SN_2013/N4E/Lista N4E-80.pdf]

CAVIDADE N4E-0080

Classe	Ordem	Fam/Outros	Gên/Outros	Espécie	S-U	U-S	Zon.
Arachnida	Acari	Cunaxidae		sp.1		X	P
Arachnida	Acari	Prostigmata		sp.49	X		E
Arachnida	Acari	Rhagidiidae		sp.2		X	P
Arachnida	Acari	Sphaerolichidae		sp.1		X	P
Arachnida	Amblypygi	Charinidae	*Charinus*	sp.1	1		E
Arachnida	Amblypygi	Phrynidae	*Heterophrynus*	*longicornis*	1		P
Arachnida	Araneae	Caponiidae		jovem	X	X	P
Arachnida	Araneae	Corinnidae		jovem		1	P
Arachnida	Araneae	Ochyroceratidae	*Ochyrocera*	sp.1		X	P
Arachnida	Araneae	Ochyroceratidae	*Ochyrocera*	sp.3	X	X	E/P
Arachnida	Araneae	Oonopidae	aff. *Xyccarph*	sp.1		X	E
Arachnida	Araneae	Palpimanidae		jovem		X	E
Arachnida	Araneae	Pholcidae	*Mesabolivar*	*aurantiacus*	X		
Arachnida	Araneae	Theridiidae		jovem	X		E
Arachnida	Opiliones	Escadabiidae		sp.1	X	X	P
Arachnida	Palpigradi	Eukoeneniidae	*Allokoenenia*	sp.1		X	P
Arachnida	Pseudoscorpiones	Bochicidae		sp.1	X		E
Arachnida	Pseudoscorpiones	Cheliferoidea		sp.1	X		E
Arachnida	Pseudoscorpiones	Chthoniidae	*Pseudochthonius*	sp.1		X	P
Arachnida	Schizomida	Hubbardiidae	aff. *Rowlandius*	sp.1		X	E
Chilopoda	Scolopendromorpha	Scolopocryptopidae	*Newportia*	sp.1		1	E
Chilopoda	Scolopendromorpha	Scolopocryptopidae	*Tidops*	jovem		1	E
Diplopoda	Polydesmida	Fuhrmannodesmidae		sp.1	X	X	P
Diplopoda	Polydesmida	Pyrgodesmidae		sp.1	X	X	E/P
Entognatha	Collembola	Cyphoderidae		sp.1	X	X	P
Entognatha	Collembola	Isotomidae		sp.1	X		P
Entognatha	Collembola	Paronellidae		sp.4	X	X	E/P
Entognatha	Collembola	Sminthuroidea		sp.2	X		E
Insecta	Blattodea	Blattellidae		jovem	1	2	P
Insecta	Coleoptera	Staphylinidae		sp.1	X		E
Insecta	Coleoptera			jovem		X	P
Insecta	Diptera	Phoridae		sp.		X	E
Insecta	Diptera	Psychodidae	Phlebotominae	sp.	X		P
Insecta	Diptera	Tipulidae	Tipulinae	sp.	X		
Insecta	Heteroptera	Cydnidae		sp.	X		E
Insecta	Heteroptera	Reduviidae	Triatominae	sp.	1		P
Insecta	Homoptera	Cixiidae		jovem	X	X	E/P
Insecta	Hymenoptera	Evaniidae		sp.1		X	P
Insecta	Hymenoptera	Formicidae	*Odontomachus*	sp.3		X	E
Insecta	Hymenoptera	Formicidae	*Pachycondyla*	sp.2		X	E
Insecta	Hymenoptera	Formicidae	*Pheidole*	sp.2	X		E/P
Insecta	Hymenoptera	Formicidae	*Pheidole*	sp.3		X	P
Insecta	Hymenoptera	Formicidae	*Solenopsis*	sp.1	X		P
Insecta	Isoptera	Termitidae	*Nasutitermes*	sp.	X	X	E/P
Insecta	Lepidoptera	Noctuoidea		sp.5		1	E
Insecta	Lepidoptera			jovem	X	X	E
Insecta	Orthoptera	Phalangopsidae	*Phalangopsis*	sp.	1	28	E/P
Insecta	Psocoptera	Epipsocidae		jovem	X		E
Insecta	Thysanura	Nicoletiidae		jovem		X	P

Malacostraca	Isopoda	Armadillidae		sp.1	X	X	P
Malacostraca	Isopoda	Philosciidae		sp.1		X	P
Mammalia	Chiroptera	Phyllostomidae	*Carollia*	*perspicillata*	3		E
Mammalia	Chiroptera	Phyllostomidae	*Glossophaga*	*soricina*	2		E
